# “I look at my own forest and fields in a different way”: the lived experience of nature-based therapy in a therapy garden when suffering from stress-related illness

**DOI:** 10.1080/17482631.2017.1324700

**Published:** 2017-05-23

**Authors:** Ulrik Sidenius, Ulrika K. Stigsdotter, Dorthe Varning Poulsen, Terese Bondas

**Affiliations:** ^a^ Department of Geosciences and Natural Resource Management (IGN), Faculty of Science, University of Copenhagen, Frederiksberg, Denmark; ^b^ Faculty of Professional Studies, Nursing and Health Sciences, Nord University, Bodø, Norway

**Keywords:** Phenomenology, reflective lifeworld research, ICD-10 F43, supportive environments, restorative experiences, nature-like setting

## Abstract

Evidence confirms that nature-based therapy (NBT) has a positive effect on people with mental illnesses. However, there is a lack of evidence on the meaning of NBT for specific patient groups. The Nacadia® Therapy Garden was designed according to an evidence-based design process, and an NBT programme was developed. The aim of the study was to illuminate the phenomenon of participants’ lived experience of the NBT in Nacadia. Fourteen participants took part in semi-structured interviews (SSIs), and by way of reflective lifeworld research, the SSIs were analysed to identify and describe the meanings of the phenomenon. The essence of the phenomenon was found to be a process of adopting a searching approach to NBT and Nacadia to become familiar with the conditions. This familiarity stimulated the development of confidentiality and attachment to Nacadia. Feeling protected, safe, cared for, and not exposed was important, and motivated feelings of freedom, reduced demands, and increased the ability to access and try a spectrum of NBT activities. It encouraged participants to develop personal approaches and coping strategies to implement in their everyday lives for moving on.

## Introduction

This study is part of a larger study, which includes, among other things, a randomized clinical trial (RCT) comparing nature-based therapy (NBT) to cognitive behavioural therapy for people suffering from stress-related illnesses. The focus of this study is on participants’ lived experiences in the University of Copenhagen’s Nacadia® Therapy Garden, during a 10 week NBT programme. It is based on semi-structured interviews (SSIs) with 14 participants.

### Background

Mental disorders are one of the main challenges to public health in Europe, with around 25% of the population being affected every year according to the World Health Organization (WHO, 2013). It is considered that stress and stress-related illnesses will be one of the greatest threats to public health in the Western world by 2020 (WHO, 2005). Stress and stress-related symptoms are a major, and in recent years increasing, cause of work incapacity and sick leave in Denmark (Netterstrøm, 2014). From a medical perspective, Netterstrøm (2014) defines stress as, “[…] a state in the organism characterized by physiological responses with activation of the sympathetic nervous system, immune system and energy mobilization and mental activation due to strain of a psychological, physical, chemical or biological kind […]” (p. 14). Stress is not regarded as an illness and, therefore, the diagnosis of individuals suffering from stress is based on multidimensional stress-related symptoms (Aldwin, 2009), which are categorized in the International Classification of Diseases, 10th revision (ICD-10) (WHO, 1992). From a phenomenological perspective, people who suffer from illness, e.g. stress-related illnesses, have had their relationship with the world disturbed. Ill individuals lose their undisturbed freedom, which involves exclusion from “life” (Gadamer, Gaiger, & Walker, 1996). Dahlberg, Dahlberg, and Nytsröm (2008) describe it thus: “When we are in pain and weak, our bodies become obstacles that keep us from immediate engagement with the world. Illness alters one’s attachment to the world” (p. 44).

This study focuses on individuals with stress-related symptoms which are so severe that they have resulted in their being incapable of working. According to the Danish Stress Society (2016), there is limited evidence of efficient specific treatment interventions for stress. The most recently available advice for practitioners from the Danish Health Authorities suggests that various forms of cognitive treatment, together with initiatives such as relaxation exercises, physical activity and, eventually, job training, can help the patient to cope with stressful situations (National Board of Health, 2007). The increasing number of citizens who are incapable of working and who are on sick leave owing to stress-related symptoms puts a burden on the Danish welfare economy, and threatens the quality of life of the stressed individuals and their relatives (Netterstrøm, 2014; WHO, 2013). There is a global, national, regional, and local demand for innovative interventions to prevent and treat stress-related symptoms (European Communities, 2005; Eplov & Lauridsen, 2008; National Board of Health, 2014; WHO, 2013, 2005). The demand is directed towards evidence-based and effective treatments (WHO, 2013). Evidence-based safe and humane interventions which involve multidisciplinary professional collaboration are needed (WHO, 2013). If society could cope with the burden caused by stress, quality of life and productivity would be improved and the number of suicides reduced (WHO, 2013).

### Natural environments and elements supporting treatment

Based on the WHO’s definition of health as: “[…] a state of complete physical, mental and social well-being, and not merely the absence of disease or infirmity […]” (WHO, 1948, p. 100)., awareness of a more biopsychosocial and multispectral approach to human health has been increasing in recent decades in health science and clinical practice, as well as in the population in general (Melchert, 2015; Pearson, Field, & Jordan, 2009; Taylor & Francis, 2013; Todres, Galvin, & Dahlberg, 2007). This multispectral view facilitates more explorative research approaches regarding innovative as well as traditional types of treatment.

The increasing amount of research from several disciplines collectively confirms the fact that natural environments are a resource in relation to human health, and further acknowledges nature-based treatments (Annerstedt & Währborg, 2011; Grahn, Ivarsson, Stigsdotter, & Bengtsson, 2010; Hartig, Mitchell, De Vries, & Frumkin, 2014; Marcus & Sachs, 2014; Stigsdotter et al., 2011). However, in a review study published in 2011, Annerstedt and Währborg found that research conducted in the field of NBT is mainly based on heterogeneous diagnosed individuals. Furthermore, while they found that evidence of nature’s therapeutic effect on human health and well-being is strong, they identified a need for more research on the effect and utility of natural environments for specific patient groups. Therefore, the WHO and the European Union’s request for evidence-based and effective treatments, the need for evidence-based safe and humane interventions in a multidisciplinary professional discourse, and the need for research on NBT offered to heterogeneous diagnosed individuals form the background for the present study. The study focuses on homogeneous diagnosed individuals who are incapable of work because of stress-related symptoms, and who have participated in a distinctly described NBT programme with well-defined endpoints. The article analyses the participants’ lived experiences of the NBT intervention.

### Aim

The aim of this study is to describe the phenomenon of participants’ lived experiences of the NBT in Nacadia during the course of a 10 week NBT programme.

## Method

To increase our understanding of the NBT in Nacadia, we use a reflective lifeworld research (RLR) approach, where we discover, elucidate, and describe the general and individual life meanings of the phenomenon of NBT. This approach was developed by Dahlberg et al. (2008), and the current study is based on their guidelines, whereby the studied phenomenon should guide a dynamic methodical stance to reach a sound and broad understanding of the phenomenon in its context (Dahlberg et al., 2008). By applying this approach, this study aims to illuminate the essence of NBT in Nacadia during a 10 week treatment programme, and to describe the essence of the phenomenon and its constitutive elements. Essence is here understood in line with Dahlberg et al. (2008) as that “something” the participants experience as the phenomenon, “i.e. the phenomenon’s style, its style of being” (Dahlberg et al., 2008, p. 247). The constitutive elements are considered to reflect the meanings of the phenomenon. Together, these constitutive elements have various nuanced meanings that are present in the essence of the phenomenon (Dahlberg et al., 2008).

### Setting: NBT in the Nacadia Therapy Garden

**Figure 1.**
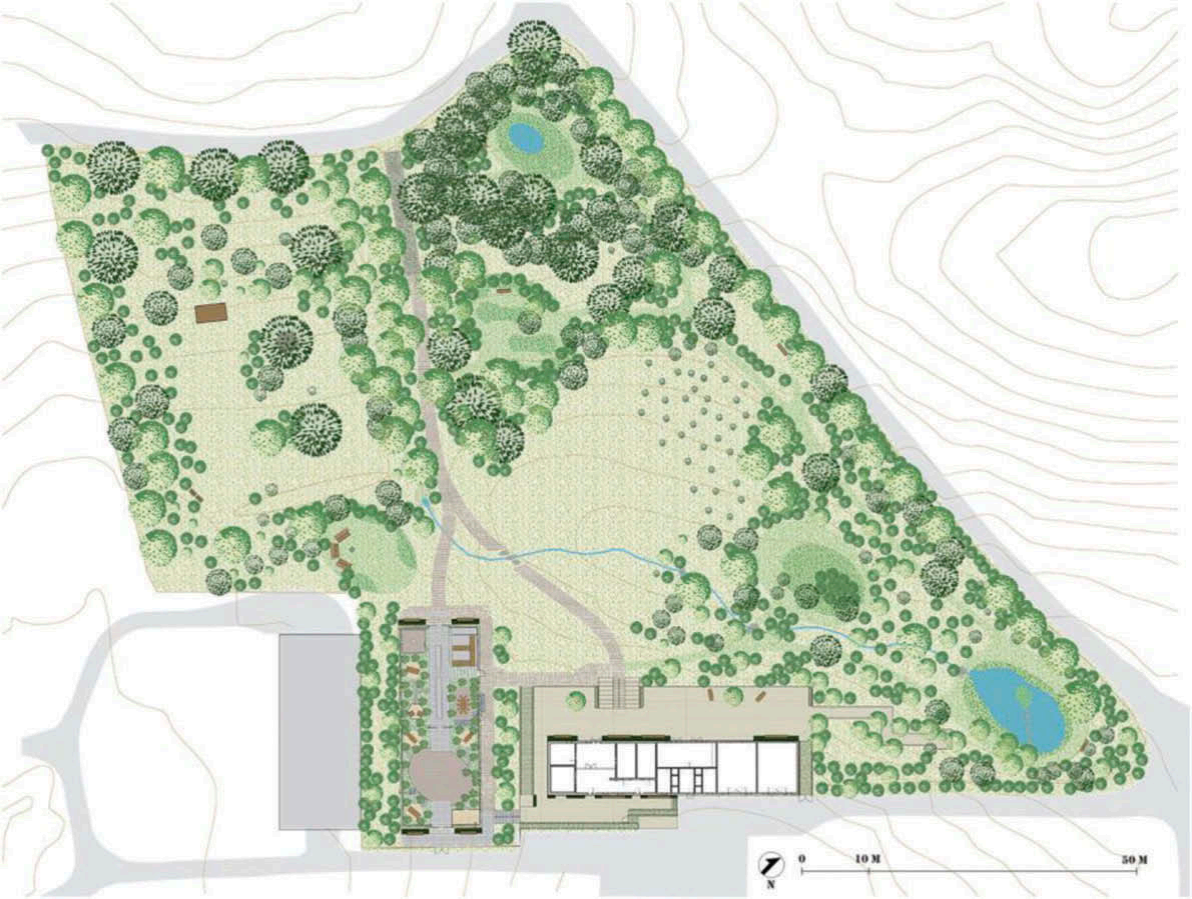
Plan of the Nacadia Therapy Garden .

In this study, a therapy garden is understood as a garden designed with the intention of actively and positively contributing to the clients’ treatment and well-being (Koch et al., 2008; Stigsdotter, 2014; Stigsdotter, 2015). The garden design is intended to fit the therapeutic process by supporting and challenging the clients by offering various nature experiences as well as providing meaningful activities all year around (Stigsdotter, 2014). The garden design and the therapy programme are closely related; thus, the design of a therapy garden is the premise of the NBT (Corazon, Schilhab, & Stigsdotter, 2011; Stigsdotter, 2014; Stigsdotter et al., 2011; Koch et al., 2008). The Therapy Garden, Nacadia (Figure 1), was specifically designed to support NBT offered to individuals with stress-related illnesses.

NBT is defined as an intervention that initiates a therapeutic process with activities that incorporate natural elements and experiences of nature in a specially designed or chosen natural environment (Corazon, Stigsdotter, Jensen, & Nilsson, 2010). The NBT programme used in Nacadia was developed as a treatment programme targeting people incapable of work owing to stress and/or stress-related symptoms. It builds on elements from NBT and mindfulness-based cognitive therapy (Corazon et al., 2010).

**Figure 2.**
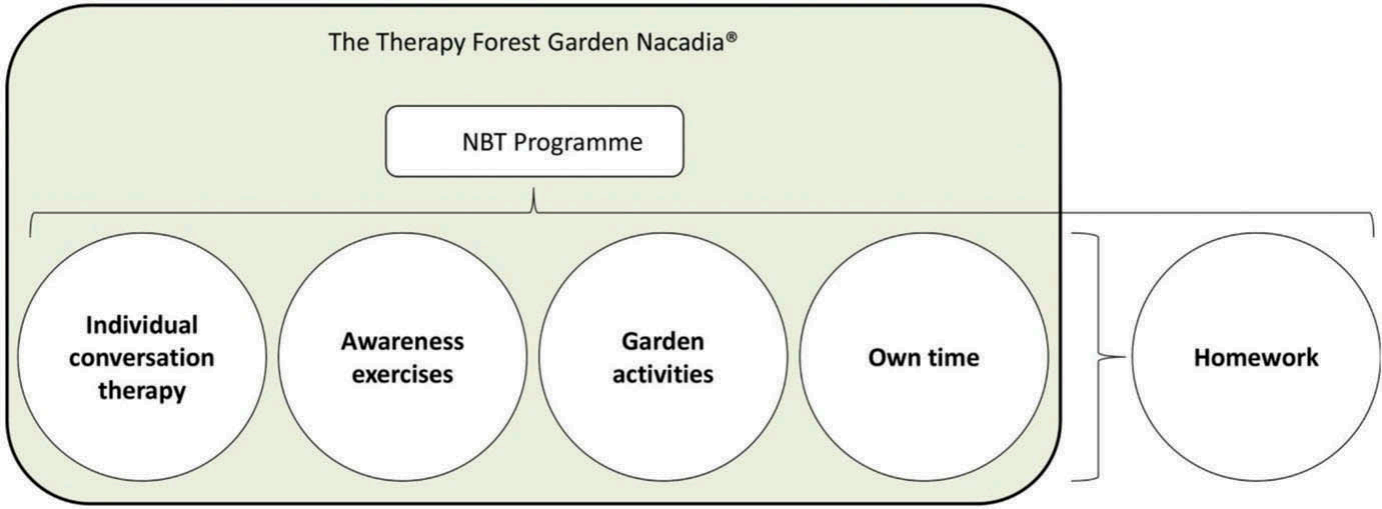
The components of the nature-based therapy (NBT) programme conducted in the Nacadia Therapy Garden.

The NBT programme in Nacadia consists of five components with an inter-supportive aim (Figure 2): (1) individual conversation therapy, which uses mindfulness-based cognitive therapy; (2) physical and mental awareness exercises, e.g. meditation and body scan; (3) garden activities, e.g. chopping wood and collecting herbs; (4) own time; and (5) homework to practise the different techniques and methods from individual conversation therapy, awareness exercises, garden activities, and own time. Although all of the NBT components are intended to apply to the whole group of participants, each component is flexible and optional, and may be adapted to the individual participant’s needs. The person–nature relations possible during the NBT in Nacadia are thought to contribute sensory stimulation and nature-related stories and symbols, and are thought to enhance the potential for relaxation and increase the participants’ experiences of being.

The NBT programme in Nacadia lasts for 10 weeks. It takes place on 3 days per week, for 3 h per day (Table I). During the current study, there was a maximum of seven participants per group and a minimum of four. The NBT is the same all year round, and the framework is the same every day. However, every week has a specific theme, in accordance with expected progress. The daily therapy was performed and managed by two authorized psychologists who are both trained in NBT. The therapists were supervised by a medically responsible psychiatrist. The garden activities were initiated and assisted by a professional gardener.

**Table 1.** Overview of the parts of the nature-based therapy in Nacadia.

Treatment	Nature-based therapy
Environment	Nature-like setting: the Nacadia Therapy Garden
Therapists	Two authorized psychologists; one assisting gardener
Treatment period	10 weeks, three times per week, 3 h per day
	96 h
Treatment set-up	Groups of up to seven participants

### Participants

In total, 42 people in seven groups participated in the NBT at Nacadia during the period from 5 August 2013 to 27 March 2015. The inclusion criteria were that the participants had to be 20–60 years of age and have one of the following ICD-10 codes (WHO, 1992) as their primary diagnosis: psychiatric diagnosis of adjustment disorder and reaction to severe stress (ICD-F43.0–9, minus 1 = PTSD). This level of stress was considered to correspond to 3–24 months of inability to work. Potential participants were excluded if they were suffering from any other significant diseases, mental disorders or social phobia, were suicidal, or had alcohol or drug misuse problems. Before their admission to the project, the potential participants were assessed to ensure that they fulfilled the inclusion criteria.

### Data collection

Owing to ethical considerations, data collection should put as low a burden as possible on the participants and, thus, the strategy was to obtain stories that were as information rich and broad as possible from as few participants as possible. Participation in the study was voluntary and the therapists aimed to ensure that the interviewees had sufficient mental capacity to attend and were willing to share their experiences. Based on the above, the therapists selected two participants from each of the seven groups whom they considered to be suitable. The 14 participants were interviewed for 20 min on average, in the second, fifth, and ninth weeks of the NBT programme. An interview guide consisting of open-ended questions was produced with topics directed at the aim of the study, and questions about the participants’ lived experiences of the phenomenon, which initially explored the participants’ general impressions with open questions, e.g. “What is your impression of Nacadia?”, which were followed by more direct questions, such as: “What do you prefer to do in Nacadia?” and “How would you describe your favourite place(s) in Nacadia?” To gain more in-depth data regarding their experiences, the direct questions were eventually supplemented by follow-up questions, such as: “Can you give me an example?” and “Can you describe your experiences from that place more?” The interviews were conducted by the first author or a research colleague. All interviews were recorded and transcribed.

Other types of data were gathered to gain more insight into the context of NBT in Nacadia, and to understand it better before the analyses of the interviews with the participants. These corroborating data were: interviews with the two therapists, observations, and participants’ private logbooks. An interview with the therapists was conducted to gain insight into their use of Nacadia during NBT, and into how they have guided participants, and observed and reflected upon the participants’ use and experiences. Observations were made using behaviour mapping (BM), a method suited to study people’s behaviour in relation to different components and features in an environment (Moore & Cosco, 2010; Proshansky, Ittelson, & Rivlin, 1970). A more in-depth description of the BM process can be found in Sidenius, Stigsdotter, and Dahl Refshauge (2015). During the BM processes, notes were taken if anything was considered valuable to reflect upon during the analyses of the interviews. The logbooks provided the participants with four pages per day for illustrating their use on a printed map and for writing their stories about their current use and experiences.

### Ethical considerations

The study followed the ethical principles of the World Medical Association’s Declaration of Helsinki (World Medical Association, 2013). It was approved by the Danish Data Protection Agency (J.nr. 2013-54-0344) and by the National Committee on Health Research Ethics (P.nr. H-1-2013-038). The participants received both oral and written information about the study and they signed to acknowledge informed consent before participation. They were informed of their right to withdraw from the study at any time and they were guaranteed confidentiality regarding the information. During the data collection and subsequent analysis and interpretation, ethical principles for qualitative studies were taken into account (Fog, 2004; Nielsen, 2003). With regard to the quotations used in this article, the sources are anonymous.

### Analysis

**Figure 3.**
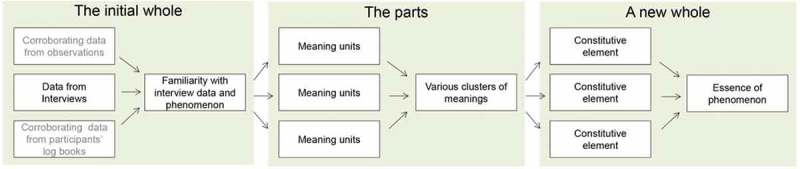
The analytical concept. The conceptual figure illustrates the process of data analysis and synthesis as conducted for current study by using reflective lifeworld research.

The interviews were analysed by the authors and the data and findings were discussed and corroborated in a dynamic and open “spiral” process. This process was in line with Dahlberg et al. (2008) and their concept of the analytical flow: the whole–the parts–the whole (Figure 3). The authors approached the participants’ narratives by restraining their pre-understandings and assumptions of the phenomenon, and further curbing their evolving understanding of the meanings as these developed. Such bridling was done with the intention of maintaining a critical reflective stance to the whole phenomenon when meanings began to evolve (Dahlberg, 2006). Initially, the interviews were listened to and read to gain an overall impression and to become familiar with the participants’ narratives and with the phenomenon (the initial whole). Secondly, parts of the text that contained meaningful content regarding the participants’ lived experiences were highlighted and set into a matrix to gain an overview of the diverse meaning units and to find clusters within these (the parts). The clusters of meanings were organized in a new matrix so that descriptive constitutive elements could develop, and an understanding of the essence of meaning and, thus, the phenomenon, could be described (a new whole).

## Findings

The rich descriptions indicate that the essential meaning of the phenomenon, participants’ lived experience of NBT in Nacadia, can be captured very well by the phrase: “I see my own forest and fields in a new way”. It is a dynamic, evolving process of exploring and developing to see and live life from new perspectives and approaches. When the participants began NBT and were new to the programme and the settings of Nacadia, they experienced a sense of uncertainty and even slight discomfort. However, the participants soon became more comfortable with the NBT programme and its procedures. Thus, a feeling of familiarity with the garden and the practical conditions grew, and a sense of belonging developed. The participants became familiar with the garden, e.g. each found locations in the garden that provided suitable shelter, which could be used to make them feel less exposed. This led to a sense of safety and freedom, which together with the sensory stimuli in the garden reinforced the feeling that Nacadia was a supportive environment. This increased the participants’ awareness of the various opportunities in NBT, and motivated more awareness of themselves and their relations with others and the world around them. The participants became more open to exploring the spectrum of opportunities in the NBT. They engaged in the NBT exercises and different activities to the extent of their individual capabilities and needs. This further encouraged them to develop personal tools, techniques, and new approaches that made them feel better equipped and gave them more courage to change and develop unique individual strategies and approaches to life for moving on after the NBT.

The following descriptions of constitutive elements will further elucidate the essence of the phenomenon: Another world of relations and environments to habituate to; Becoming more comfortable and developing a sense of belonging; Suitable shelters offer less exposure and a sense of safety and freedom; Sensory experiences reinforced Nacadia as a supportive environment; Increased awareness of destructive mindsets; Spectrum of opportunities meeting individual capabilities and needs; New approaches, more courage to change and move on.

### Another world of relations and environments to habituate to

Each participant experienced the new settings of Nacadia differently and expressed various impressions. In the groups, an overall sense of hesitant anticipation of what was going to begin was often noted. The participants experienced starting the NBT as a challenge as there was a lot of new information, etc., to deal with: “Trying something new has been a challenge for people like me. The first few times I was here, it was very new and a little dangerous. I wasn’t quite used to it”. However, the participants’ overall initial attitude to NBT was positive and optimistic, and it was viewed as an opportunity and a necessary step on the path towards becoming better able to cope with the challenges in daily life.

In the beginning, the participants usually thought that the physical environment of Nacadia was “welcoming”, “beautiful”, “fantastic”, and a “magical world of its own”. Besides the new environment of the garden, the participants also had to adjust to the new practical conditions for participating in the NBT, and whatever reorganization of daily routines that required, e.g. travelling to Nacadia three times a week, and the facilities at Nacadia, such as restrooms and locations for storing garden equipment, etc. The NBT programme was also mentioned as part of the new conditions to adjust to, and participants speculated as to whether there were any expectations, routines, and rules of which they should be aware.

The most pronounced challenge that needed to be overcome was getting to know, and getting on with, the other participants in Nacadia. In the second week of NBT, a participant said the following: “I have not completely understood the guidelines—how much we should talk to each other—why you are here, what you feel and stuff (…) It’s a little strange to walk around with seven people that you know you will be around for the next ten weeks, three times a week (…) But it is really fine”. Some were worried about the disturbances other participants might cause them. The concerns regarding other participants were not expressed as problems, but rather as a certain cautiousness towards other participants during the process of becoming familiar with how to get along with them during the NBT. Those who saw their other participants as possible risks of disturbances in the beginning, seemed to become aware of using different opportunities in the NBT setting to cope with potential disturbances later in the NBT period. One participant said: “The various offers here support both [social and solitary need]. So, when I feel good, I choose something social and when I don’t feel so good, I choose something more withdrawn where I can be myself”. All the participants expressed different social needs and capabilities, and that these varied from time to time. Although it took several participants some time to develop strategies to cope with the presence of others, and to get along with the other participants, most participants adopted suitable tactics to satisfy their varying individual social needs during the 10 week NBT.

### Becoming more comfortable and developing a sense of belonging

The nature-like environment soon gave the participants peace of mind and allowed them to wind down: “When you are here, it is a world apart. It works quietly and I decrease in pace and become calm”. Such a feeling of calm was experienced by some as physical and mental comfort. Even at the beginning, several participants expressed experiencing calmness of mind and body: “… you find such calmness and you can feel your body—reflect, become aware, feel your body, calm down totally”.

The experience of being cared for was also mentioned and seemed important, as one described: “There is a tremendous care for one another and from the therapists. It’s a five star hotel stay. There is so much care”. Another participant explained how she felt that the therapists helped the participants to become familiar with using NBT components efficiently for personal development: “The therapists make you aware of what is going on in your mind, thought patterns and help to change your behaviour, the way you think, tools to approach tasks differently. It’s quite effective”.

The care, support, and guidance, and the well-organized and well-maintained practical conditions—the experience that things were under control in Nacadia—seemed to be important in the participants finding comfort, safety, and peace of mind.

Some participants stated that they found they were comfortable with some features in the garden, and how they became familiar with and connected to the garden by developing certain routines in the environment: “I have different shrubs and trees that I keep an eye on to see how they are doing (…) I have developed a connection to the place. And I’m happy every time I go there. It has been a revelation to see and follow”. Such a relationship was described by another as “belonging”, and seemed to be strengthened by the fact that participants had the opportunity to have an influence on the garden during the activities, e.g. during maintenance, planting, and harvesting, and were able to follow the subsequent developments resulting from their activities. This gave them a feeling of responsibility for the garden, which motivated a feeling of attachment and ownership: “Now I feel that this garden is also my garden. I feel that I have taken some responsibility for the maintenance of the garden. I have a co-responsibility, which I like. It’s a place where I have put some heart, a lot of thought, and gained enormously in return”.

### Suitable shelters offer less exposure and a sense of safety and freedom

The perception of being less exposed and being sheltered from the outer world in the fenced-off garden seemed to be an important part of creating a safe environment where the participants could begin to use and explore different NBT exercises and activities. Most participants described experiencing a feeling of freedom to choose, explore, and use NBT in accordance with their personal needs at any time while in Nacadia, e.g.: “We are allowed to approach it like children, try to think, what was it like to be a child, to explore and be curious”. The experience of being as free as a child seemed to be partly stimulated by a feeling of having no external obligations and feeling a diminished level of external expectations or demands, which was strengthened by the support and care of the therapists and peers. This freedom gave the participants space to explore, select and try, or not try, activities: “For the first time, I haven’t done any activity, and it was also great. I didn’t feel guilty about it. The others are buzzing around with bees, and streams, and all possible tasks. I just sat there. I have never done that before, but it was actually OK”. Thus, the freedom made the participants more aware of how different activities and locations in Nacadia suited their personal current needs, e.g.: “I’ve been to different places to see how they affect me. In the beginning, I went for something visual, something that was pleasant. But now I prefer something that feels good and it’s very much the senses that decide”.

### Sensory experiences reinforced Nacadia as a supportive environment

As time passed, Nacadia was experienced as an overall frame for the different components of the NBT. One participant explained: “It’s the combination, the place is just the frame around what we do during those awareness exercises and meditation. It is probably the individual components that work the best, but the whole, the whole also makes a good framework”. He explained how he had come to understand NBT as being comprised of mutually supportive components. He thought that the garden represented a frame for NBT, which strengthened the various NBT exercises, as he added: ”It would be something else if we sat in a room in the middle of the city—it wouldn’t be as good as here”. It appears that the nature-like environment was experienced as an overall supporting framework in the sense that it made the exercises, activities, and therapeutic conversations more accessible. The overall settings of Nacadia made the participants feel calm, safe and, therefore, more receptive to the NBT: “One is attentive and somehow focused on something or other in nature. That is probably it, opening up all your senses and beginning to listen and smell and see much more—being more aware of the present”. An example of nature’s role as a supportive setting is illustrated by a participant’s description of why he preferred his individual conversation therapy sessions to be conducted as “walk-and-talks”: “So we have done that every time. It’s great to walk around and talk—more natural things and just to go for a walk and talk”. In this case, the nature-like setting facilitated conditions which made it possible for him to choose a set-up that made it easier for him to talk in depth about personal matters. Another participant suggested that the garden settings made a walk-and-talk session a positive and motivating experience because nature presented scenes and offered various experiences which eased any existing tension. Nature provided sensory stimuli which led to both physical and mental relaxation that stimulated increased responsiveness and greater openness to opportunities, and a greater ability to accept.

### Increased awareness of destructive mindsets

On several occasions, participants shared stories of how they gained a new level of self-awareness. Some participants said that during the NBT activities they had become aware of falling into destructive patterns of mindsets with feelings of pressure to perform and deliver, and/or a sense of competing. They became aware that such patterns and corresponding ways of acting were identical to the mindsets and working habits they had often experienced during everyday situations, e.g. at work, and recognized such patterns as being destructive and causing stress.

During NBT, they became increasingly attentive to those patterns and increasingly aware of what triggered them. A participant shared her experience with the NBT activities: “I experienced that I had some performance anxiety. I had to make a beautiful bouquet though I’m not a florist—I had to do it on time, and what were the requirements? Many of those patterns (…) follow you in nature, but you can then let go. So I caught myself in it”.

Participants further shared experiences of stepping out of their habitual mindset and usual considerations by sitting alone during their own time, and shared how such experiences could be stimulated by certain physical conditions and the atmosphere at specific locations in the garden, e.g.: ”It’s absolutely fantastic. It’s both the light and also those tall, tall trees. It’s enclosed, but you can still look out. You feel a bit in in awe when you walk in there. You feel quite small and insignificant when you sit there—but that is also very nice sometimes”. It led to a sense of something beyond human scale, role, and measures, and gave the participants an opportunity to consider themselves and their situation from a new perspective and to let go of normal thinking and self-judgement. In this way, many participants used the nature-like settings in Nacadia as a catalyst to step out of their habitual mindset, motivate self-reflection, and gain a new self-awareness.

The increased self-awareness seemed to give the participants strength to challenge themselves and to act upon their current needs. A participant exemplified how she acted in accordance with her current experience of bodily disturbance one day: ”I was so restless in my body—a bit frustrated and a little tired and annoyed. So I just sat here and observed. There was no wind and absolute quiet—this peace slowly seeps into the body. So I had no need to get rid of energy in that way”. Previously, this participant said that she had a habit of being very physically active when she felt restless. This exemplified how participants during NBT became aware of themselves and new alternative approaches to coping with bodily and mental needs.

### Spectrum of opportunities meeting individual capabilities and needs

It is important to note that not all participants on all days found it easy to find suitable places to go or activities to do. On days when their minds were in turmoil, a few participants expressed experiencing difficulties in finding a location suitable for their needs, although this was the exception rather than the rule. It seemed that as the NBT progressed the participants increasingly used the opportunities in Nacadia to act more consciously in accordance with their current needs: “In the beginning I thought: ‘I should do something’. But the therapists have repeatedly said: ‘Try and feel what you want right now, or can handle right now, need right now’”. When the participants described the different opportunities, they referred to “opportunities” as various activities, locations, and choices within them, e.g. whether to interact, be physically active, be physically or mentally challenged, and the extent of the sensory stimuli. In general, the participants were able to choose activities and locations that suited their individual current needs: “There are plenty of opportunities to find whatever suits each of us, otherwise, you just have to find it”.

The participants’ mood, capabilities, and needs continuously changed. Furthermore, each participant’s experiences were independent of other participants’ experiences. For example, towards the beginning of the NBT, one participant (A) had a lot of energy and chose a physically challenging activity: “I also like to exert myself a bit, to get a little sweaty, right? So I chose the scrubbing brush and scrubbed algae off the bench”. On the same day, another participant (B) said that her energy level was low and she was not in a good mood and, therefore, chose a less challenging activity: “I have just had some days where I have pushed myself a bit too hard. And then I thought, I should not do anything hard”. Participant B also chose to spend time inside the greenhouse because of her sensitivity to the weather conditions. In contrast, participant A stayed outside and did not mention the weather at all. Both participants expressed that they had the opportunity to meet their respective capabilities and needs on that specific day.

As well as experiencing contrasting needs, participants experienced contrasting meanings. On the same day, two participants explained their different reasons for choosing and not choosing, respectively, to do a particular activity (placing wood-chips next to a hammock) and how they experienced contrasting meanings linked to this: “Today I didn’t have as much energy as I have had previously. And luckily, not so much needed to be done. So that fitted well together. My mind is a bit restless”. Because the participant was experiencing disturbing thoughts at the time, he preferred to perform repetitive, physically demanding activities, while he avoided activities that were more mentally challenging. The other participant explained how she chose another activity that seemed meaningful to her: “Putting wood chips under the hammocks—that I opt out of because I think it’s not important. I like it when things have a purpose”. Despite individual differences, both of the participants found opportunities that suited their specific contrasting needs, which gave them individual, meaningful, and supportive experiences.

### New approaches, more courage to change and move on

Having become familiar with NBT and having seen and/or tried NBT activities, the participants gave more attention to considering how to choose and approach activities that suited their current physical and psychological capabilities. One explained: “Deliberately I didn’t choose tasks which I knew would be very physical activities because I’m really tired. Activities that require too much physical strength I just couldn’t grasp. I reject those where you need instructions and where it gets complex”. The general approach to the activities became increasingly explorative in character, with the aim of identifying activities that felt most meaningful and beneficial to the individual participants. One of the participants explained how he had obtained personal benefit from the NBT activities with his unique approach to gaining peace of mind: “It is a rhythm in the work—it may lead to peace of mind with a rhythm in the work. And it’s also a pleasure to see something being built up. To have a result”. Another shared how he had deliberately selected a garden activity which he had previously avoided because he had previously fallen into a negative habitual mindset during the activity. However, towards the end of NBT, he felt equipped to challenge himself and attempt a new approach rather than his habitual mindset and working habits during the activity: “I have a tendency to go in and make things more efficient and do things as quickly as possible and just finish. But that’s one of the things I’m working on, there’s no reason to rush things. So it’s just nice, nice and easy—and that’s something that has probably changed very significantly in me”.

Participating in the NBT made several participants aware of new alternative approaches to daily tasks and habits. Almost all of the participants talked about how they had become aware of such new approaches in the safe and protected environment during NBT. One participant was fearful of tall trees and dark and enclosed rooms. However, by the end of the NBT, she explained that she had developed more courage and had gained tools to challenge her own fear of tall tress by exposing herself little by little to the fear in Nacadia. This demonstrated how she had become more self-aware and capable of acting in line with a new constructive approach. Her next goal was to gradually expose herself to dark and enclosed rooms as a further step in her personal development. Another participant explained how she had started to reflect on her usual approach to working tasks during activities in Nacadia: “Whatever garden task it is, I learn something from it. It may be like a trip back in time, where I can see how I handled things before: ‘How hard can it be?’, but then all of a sudden I can recognize that there is also another way of approaching things”. Another participant talked about how she had become more aware of her routine and less constructive approach to different tasks in her daily life, and how she then began to use a different approach: “I look at my own forest and fields in a different way. I’ve become aware that there is a little more: ‘it has a different colour today’ or ‘the light is falling in a different way today’. I have also used it at home with all the household chores you have: ‘How important is it that I do the hoovering today? Do I feel like doing it now or should I instead sit down with a book?’ So I am more present”.

During some interviews that were conducted 1 year after NBT in Nacadia, some participants shared their insights into how they have implemented the therapeutic tools in their daily lives. For example, some participants took their usual walks in their local green environments, such as forests, parks, and neighbourhoods, but at a slower pace to find new sensory experiences there, which helped them to achieve a more aware state of mind, as they had learned during NBT. One participant explained that he had installed a small fountain in his garden which made calming rippling sounds like those he had experienced from the stream in Nacadia. The experience of the sound of the water helped him to achieve a calmer state of mind during meditation or relaxation at home. He also said that after NBT he kept a pinecone in his desk drawer at work. If he had too many disturbing thoughts, he would pick up the cone, close his eyes, feel its texture in his hands, and listen to the crackling sounds and smell the scent of resin from the cone. Experiencing these sensory stimuli for a few minutes helped him to attain a calmer state of mind and forget the disturbing thoughts.

Almost all of the participants expressed experiencing development and general improvement in their well-being during the course of the NBT, and almost all of them expressed experiencing changes in their daily life, e.g.: “I think that things are gradually improving. And I’m becoming happier. Everything is easing a little and daily life is becoming easier. Those ordinary everyday things that before were completely overwhelming, they almost get done by themselves now”. One participant did not directly communicate an improvement in his general well-being. However, along with his peers, he stated that he had gained useful tools to use again in the future. Although the participants’ stories differed, all of them talked about how NBT had made them aware of new approaches for moving on in life: “It’s as if we’ve learned some kind of strategy to how life can be lived in a slightly different way than before we came in here or before we became ill. And we have probably learned, through what we have done, and from each other and the therapists—and perhaps in fact also from nature”.

## Discussion

### A wholeness of environments, relations, individuals, and opportunities

The participants seemed to experience the constituent parts of NBT in Nacadia: the nature-like settings, the garden activities, the exercises, the therapists, the peers, and themselves as individuals, to interact as “wholeness”. The different constituents offer flexibility and mutually supportive experiences that provide the participants with an inner freedom to explore, find, gain, and develop. This diverse wholeness may be interpreted as being a prerequisite for the participants to become more aware of themselves as a “human whole”. It enables the participants individually to find what specifically supports and stimulates them in their own time and pace, which motivates them to feel physically and mentally relaxed and open to the NBT components and those sensory experiences that support positive associations, memories, and reflections. This is in line with the original overall salutogenic ambition of the project: to emphasize what is strong and healthy within each individual (Antonovsky, 1996). Furthermore, it seems to be consistent with the more holistic understanding of human beings and human health found in contemporary nursing and health science (Melchert, 2015; Pearson et al., 2009; Taylor & Francis, 2013; Todres et al., 2007). The understanding and the experiences of wholeness can be elaborated from a lifeworld perspective, which suggests that the body is constantly perceived and perceiving so that the surrounding world becomes meaningful as it is experienced through the body as it carries out our living actions (Dahlberg et al., 2008; Merleau-Ponty & Smith, 1996). This explains how humans experience and relate to contextual world situations in order to understand and make meaning of them, and how the mind, body, and the surrounding world make up a lived relationship of wholeness. From a lifeworld perspective, our attachment to the surrounding world is disturbed when we become ill (Dahlberg et al., 2008). Therefore, when a participant increasingly experiences himself or herself, the NBT settings, and the constituents as a whole during NBT, it can be seen as a sign that he or she is in a state of recovering, as his or her “body” apparently has developed to become stronger and better able to deal with and connect with the surrounding world.

### Experiences of Nacadia

The participants described an increasing attachment to Nacadia, and terms like “belonging” and “ownership” were used. Such developed emotional bonds to Nacadia can be characterized as positive place attachment (Manzo & Devine-Wright, 2014). Place attachment can phenomenologically be defined as “any environmental locus in and through which individual or group actions, experiences, intentions, and meanings are drawn together spatially” (Seamon, 2014, p. 11). This definition covers the various interdependent perspectives of participants’ stories and reflections of their lifeworld experiences of NBT in Nacadia.

When the participants described their experiences of being in Nacadia or at certain locations in the garden, they used the exact terms, or very closely related words, used by Kaplan (1995) to characterize a restorative environment: fascination, being away, extent, and compatibility. These components are related to attention restoration theory (ART) (Kaplan, 1995; Kaplan & Kaplan, 1989), according to which the restorative effect is thought to occur as the environment facilitates a relaxed state of mind which is dominated by effortless spontaneous attention focused on the constantly changing fascinating, but calm, stimuli, which provides a respite from directed attention (Kaplan, 1995). Based on the participants’ experiences and use of terms, Nacadia could be characterized as a restorative environment according to ART.

### Freedom to develop

Once they had become familiar with the NBT, all of the participants felt that they had the space and freedom to explore, experiment, and develop. The different components of the NBT programme gave the participants the opportunity to discover which components (“therapeutic tools”) suited them best; something which could change during the course of the NBT. At the same time, they discovered new approaches to the different NBT components. The participants all expressed having found their personal approach, routines, and preferences regarding the components of NBT, with the aim of gaining unique tools for “moving on”. Flexibility seems to be a strength of NBT. All participants experienced a sense of freedom and flexibility regarding NBT so that it could be adapted to their current personal capabilities, needs, and preferences.

The safe and free framework of the nature-like setting seems to have made the various exercises, the garden activities, and the conversational therapy more accessible, and made it possible for all participants, regardless of their background, to explore and develop their unique therapeutic tools.

In addition, the combination of different physical and mental NBT exercises and activities may strengthen the grounding of the participants’ experiences gained during NBT. In the literature on embodied cognition, learning (in this case experiencing the NBT tools) is grounded better if the gained experience is embodied (Corazon et al., 2011; Sutton & Williamson, 2014). This means that a concrete action that is experienced through the associated bodily sensations and mental associations will be grounded more firmly, so that eventually the body may be a cue for triggering personal embodied memories of the experiences (Sutton & Williamson, 2014). Thus, an NBT activity that a participant feels is beneficial is thought to be better remembered and recalled because it is experienced both cognitively and bodily and, thus, is embodied and grounded through a broader range of sensations and associations. Eventually, the embodied experiences can be more easily transferred from the NBT setting to everyday life situations.

### Participants’ development

Throughout the 10 weeks of NBT, the participants expressed experiencing varying levels of physical and mental capabilities, which were reflected in their behaviour and choice of activities. During the 10 week timespan of NBT in Nacadia, the participants’ capabilities seem to fluctuate, and even decrease in a few cases. However, the participants experienced increased self-awareness of their own current capabilities so that they could act more in accordance with them. This may be explained by the participants becoming more grounded in their bodily sensations, thus raising their awareness of their bodily needs. Mehling et al. (2012) describe body awareness, when beneficial to health, as being mindful, non-judgemental awareness and a sense of self, grounded in physical sensations in the present moment. Such increased self-awareness may have equipped the participants to be more conscious of acting, rather than neglecting, when they experienced low capabilities. Therefore, low capabilities may be experienced and expressed towards the end of an NBT programme as often as at the beginning. Thus, the development of participants’ well-being can be said to have increased linearly during NBT, despite experiencing low capabilities towards the end. This development may be interpreted as an improvement in the participants’ executive function, which refers to independent and purposeful behaviour, and a person’s capacity to prioritize, plan, and carry out a duty (Diamond, 2013; Jurado & Rosselli, 2007). A Swedish study at the Alnarp Rehabilitation Garden by Pálsdóttir et al. (2014), focusing on clients’ (who were suffering from severe stress or depression) experiences of nature-based rehabilitation in relation to the role of the natural environment, also found a general improvement in their executive functions.

### Strengths and limitations

Participants were recruited from an RCT group of participants for the 10 week NBT, which limited the sampling. However, as we wanted to understand the complex phenomenon of NBT as fully as possible, we needed to collect rich descriptions to help us to gain an understanding of life as it is lived by the participants who are experiencing the complex phenomenon.

Thus, all the informants had participated in NBT, and we successfully included informants with different background characteristics (gender, age, and occupational, marital, and health status), which contributed to the variation.

We were aware that some of the informants might be less capable of sharing their story in depth on certain days owing to their stress-related symptoms. Furthermore, we also had ethical considerations in mind—that we should place as low a burden as possible on the informants. The sample size may be considered small in relation to the aim of exploring a complex phenomenon; however, the informants were interviewed three times during the NBT and were broadly representative with regard to background characteristics, while we found their narratives to be very rich.

The SSIs were conducted by the first author or a colleague, who had a similar academic background in landscape architecture and therapy gardens and, thus, similar pre-understandings of the phenomenon. Before conducting the interviews, we discussed and arrived at a common understanding of the content and style of the interview manuscript to ensure that the topics of the aim of the study were sufficiently explored.

A possible limitation of the study was that the SSI guide had to be brief and focused on the aim of the main study, because of the participants’ symptoms and in accordance with the code of ethics. At certain times, some participants were not able to express and share their experiences owing to their stress-related symptoms. However, if a participant was unable to fully answer in-depth questions, it was possible for the interviewer to adapt the SSI guide to reflect the participant’s capabilities, while at the same time staying focused on the relevant topics of interest for the study. Despite the fact that some of the interviews were brief and the results were less information rich than others, all the interviews were useful and the descriptions were detailed enough to give the interviewer and researchers a good insight into the participants’ perspectives on their lived experiences of NBT.

Dahlberg et al. (2008) describe the need for a variety of complementary methods when dealing with complex phenomena from a lifeworld approach to obtain rich data to counter the ambiguity of people. Further, they stress the importance of carefully selecting methods to obtain the aimed-for knowledge accurately and effectively (Dahlberg et al., 2008). BM, the participants’ written logbooks, and the interviews with the therapists were applied to obtain additional data to support the interviews with the participants. The BM process gave the researchers a good insight into how the garden was being used. During the observation process, the first author noted the participants’ actions and behaviour, and gained a preliminary understanding which facilitated further exploration during the analysis of the participant interviews. The participants’ logbooks further strengthened the researchers’ idiographic understanding of the individual participants before analysing the various narrative styles. Such a mix of methods is time consuming, but may be considered a strength as it allows the researchers to analyse the participants’ narratives in corroboration with their background knowledge of the phenomenon acquired from other sources, and to enrich their understanding of the studied case, the individual participants from an idiographic approach, and the group of individuals’ lived experiences of the setting.

A strength of the study is related to the research team. Two of the authors are landscape architects who have solid knowledge of therapy gardens and how physical environments may interrelate with NBT. They also had a pre-understanding of Nacadia, although they strived to constrain their preconceptions in accordance with the phenomenological RLR approach. This strengthened the researchers’ understanding of the participants’ narratives of their experiences. To enhance the reliability of the results, two authors from physiotherapy and nursing science took part in the study in order to contribute to and enrich the understanding of the phenomenon and the participants’ stories. The author from nursing science had no previous connection to the garden or NBT, although she had vast experience of conducting phenomenological research.

This study contributes deeper knowledge of patients’ varied and sometimes paradoxical lived experiences of an NBT intervention. Together with the RCT, it adds to the understanding of NBT interventions.

## Conclusion and implications for practice

This study contributes new knowledge of participants’ lived experiences of 10 weeks NBT in the Therapy Garden, Nacadia, through a dynamic interplay of methods, researchers, and participants. We have explored the experience of NBT in a nature-like setting for people incapable of working owing to stress-related symptoms. By conducting an in-depth RLR analysis from a lifeworld phenomenological approach, we have learned that NBT in Nacadia is experienced as a process of habituating, becoming familiar and comfortable, and exploring and developing within the NBT conditions and settings. The participants experienced a feeling of being shielded and cared for in a supportive setting; this gave them a feeling of safety and freedom, which facilitated physical relaxation and peace of mind. Being comfortable with the NBT components made the participants more aware of selecting activities in accordance with their current individual capabilities and needs. The participants seemed to gain more courage to explore new approaches for making changes, and applying and implementing gained coping strategies from NBT in everyday situations for moving on.

The participants’ overall development can be described as an increasing self-awareness leading to an improved ability to act in accordance with their current experienced bodily and mental capabilities and needs.

The flexibility of the NBT programme and the spectrum of opportunities in NBT facilitate the positive development of all participants at their own pace, in line with their unique personal preferences and previous and current experiences of life.

This study has cross-disciplinary value for the field of landscape architecture, and for medical, nursing, and therapeutic sciences and practices. Landscape architects may use the results to guide evidence-based design processes for supportive environments, while physicians, nurses, and therapists may gain insight and inspiration for NBT procedures from the patients’ perspective. Furthermore, the findings motivate cross-disciplinary collaborations between designers and clinicians with regard to NBT.

When NBT interventions or practices are developed, it is important to keep in mind that it must be possible for each participant, regardless of personal background, to find suitable shelter to become comfortable, and feel safety and a sense of belonging at a level that allows them the freedom to discover at an individual pace. Furthermore, it is of high importance that an NBT intervention or practice offers a spectrum of opportunities of activities, experiences, environments, and choices to meet the individuals’ current fluctuating capabilities and needs.

Scientifically, the findings may inspire new studies in the field of evidence-based health design within landscape architecture. For example, it may be interesting to interview the participants not just during NBT, but also after 6 months and 12 months. It would be useful to learn more about the participants’ reflections on NBT and how they implemented what they learned from NBT in their daily lives in the long term.

As phenomenological RLR, this study describes the lived experience of the participants and what they consider to be meaningful, but has no focus on causality or effect. Therefore, additional studies which apply different methods are needed to gain a fuller understanding of NBT in Nacadia and develop more knowledge of NBT for people who are incapable of work owing to stress-related symptoms.
